# Mulberry Bark Alleviates Effect of STZ Inducing Diabetic Mice through Negatively Regulating FoxO1

**DOI:** 10.1155/2019/2182865

**Published:** 2019-01-21

**Authors:** Fan Qiu, Jiang Wang, Hua-Yu Liu, Yu-Qing Zhang

**Affiliations:** School of Biology and Basic Medical Sciences, Soochow University, RM702-2303, No. 199, Renai Road, Dushuhu Higher Edu. Town, Suzhou, China

## Abstract

Dysfunction of insulin secretion and hyperglycaemia were commonly found due to damaged *β* cells of pancreas. In our previous research, it was found that mulberry branch bark powder (MBBP) was effective in treating diabetes in mice which were induced by STZ and high fat diet. The present study was designed to evaluate the protective effect of MBBP on STZ-induced *β* cells injury and investigate underlying mechanisms. By preventive administration of branch bark powder, the damage caused by STZ injection was found to be alleviated. In MBBP feed groups, pathological weight loss was inhibited, fasting blood glucose was controlled, the incidence of diabetes decreased, and blood lipid level and antioxidant capacities were restored. The PI3K/AKT/FoxO1 signal pathway was found to be activated by key proteins expression and gene testing. In liver, the increased PI3K and phosphorylated AKT, the phosphorylated, and inactivated FoxO1, which regulates the expression of gluconeogenic gene and explains the effect of relieving insulin resistance of MBBP. Therefore, the MBBP improves the tolerance of pancreas to the toxicity of STZ involving the PI3K/AKT/FoxO1 signalling pathway.

## 1. Introduction

Streptozotocin or streptozocin (STZ) is a naturally occurring alkylating antineoplastic agent that is particularly toxic to the insulin-producing *β* cells of the pancreas in mammals. It is used in medicine for treating certain cancers of the islets of Langerhans and used in medical research to produce an animal model for hyperglycemia, as well as type 2 diabetes or type 1 diabetes with multiple low doses [1].

As a glucosamine-nitrosourea compound, STZ is similar enough to glucose to be transported into the cells by the glucose transport protein GLUT2 which is expressed relatively high in *β* cells and causing alkylation of DNA [2]. The other toxic effect of STZ involves activation of inducible NO-synthase, increase in NO concentration [3], and increased H_2_O_2_ generation [4]. A single dose of 50 mg/kg body weight in a rat will cause necrosis of *β* cells followed by *β* cells loss and atrophy of the islets [5].

Mulberries (*Morus alba* L.) are a deciduous tree in the family of* Moraceae* and are widely cultivated in China, Korea, India, Brazil, and others. The leaves of mulberry are valued as the primary food for silkworms, supporting the silk industry for centuries. The branch of cultivated mulberry is a one or two year branch and used as fodder in agriculture.

More importantly, mulberries have medicinal properties and have been used in China for a long history. According to the Compendium of Materia Medica records, mulberry had been used in “Xiao ke” (diabetes) in ancient China. Modern research has proven that the leaves and fruits of the mulberry tree have antidiabetic bioactivity [6–8]. The chemical components of* Morus alba* have been clearly listed in several reviews [9–11].

The mulberry has rich bioactive compounds in its secondary metabolites, such as alkaloids, phenols, polysaccharides, and flavonoids. With flavonoids as major constituents, mulberry leaves possess antioxidant, glucosidase inhibition, antihyperlipidemic, antiatherosclerotic, and antiobesity activities [12]. Besides mulberry leaves, more studies have suggested that branch bark also has various biological functions, such as hypolipidemic [13], hypoglycemic [14, 15], antioxidant [16], anti-inflammatory [9, 17], and antitumor [18, 19] functions.

In our laboratory, we studied the extraction of active components such as polysaccharides [20, 21], 1-deoxynojirimycin [22], mulberroside A [19], and morusin [23] from mulberry branch bark. The results showed that the extract of mulberry branch bark has bioactivities of antioxidation [19], hypoglycaemia [24], hypolipidemia, and anticancer [25]. In addition, our group investigated the interference effect of oral administration of mulberry branch bark powder (MBBP) on the incidence of type 2 diabetes induced by STZ in mice [26].

The aim of this study was to evaluate the protective properties after the preventive administration of branch bark powder in mice which were against *β* cells injury induced by STZ and examined whether the activation of PI3K/AKT/FoxO1 signalling is involved in the protective effect of MBBP treat.

## 2. Materials and Methods

### 2.1. MBBP Preparation

We followed the methods of Liu et al. (2016) [26], and the branches of the mulberry (HuSang 32, a cultivar from* M. multicaulis* L.) were obtained from the mulberry garden of Soochow University, Suzhou, China, in November 2016. The bark, which was peeled from the mulberry branches, was dried at 100°C for 2 h, pulverized into powder twice, and passed through a 100-mesh sieve. The powder was weighted and mixed with standard diet to get 2.5%, 5%, and 10% MBBP diets for mice.

### 2.2. Animals

Male ICR mice (SPF) (4 weeks old, 18–22 g) were obtained from the Experimental Animal Centre of Soochow University. All animal experimental protocols used in this study were approved by the Animal Ethics Committee at Soochow University (number of animal license: 201603A255). The mice were raised in standard experimental conditions of humidity at 50%–80%, temperature of 18°C–25°C and 12-hour light/dark cycle. During the experiment, the mice could freely obtain standard laboratory chow and standard laboratory water.

### 2.3. Drug Administration

Drug administration was also followed the methods of Liu et al. (2016) [26], the mice were allowed to adapt to the laboratory environment for one week, and then a total of 50 mice were randomly divided into 5 groups: normal group with standard feed, model group with standard feed, 2.5% MBBP feed group, 5% MBBP feed group, and 10% MBBP feed group. The mice were fed their individual diets for 2 weeks. On the 14st day, the mice were given single tail-vein injection of freshly prepared citrate buffer (pH 4.5) solution of STZ (100 mg/kg) [27], except for the normal group (STZ [S0130] was obtained from Sigma-Aldrich Fine Chemicals, United States.). After injection, all mice continued to be fed as above.

### 2.4. Fasting Blood Glucose, Serum Insulin Levels, and Organ Coefficient

Fasting blood glucose (FBG) was measured by tail-vein sampling using a glucose meter from Johnson & Johnson Medical (Shanghai) Ltd. (China) on day 7 after STZ injection. Then the mice were killed under anaesthesia. The blood was collected, and serum was prepared by centrifuge (3000 rpm, 15 min). The serum insulin levels were determined by Insulin Assay Kit (Nanjing Jiancheng Bioengineering Institute, Nanjing, China). The mice were dissected, and then liver was weighed. The ratio of tissue weight to body weight was calculated.

### 2.5. Histological Examination and Immunohistochemistry Assay

Fresh mice pancreas was quickly removed, rinsed with cold phosphate buffered saline, immersed in 4% paraformaldehyde solution at pH 7.0, and embedded in paraffin. Slices of 5 *μ*m thickness were cut. After removing paraffin with xylol, the sections were stained with hematoxylin and eosin (H&E) and examined by optical microscope (400×).

To observe the immunohistological staining of insulin, the pancreas samples were soaked in 4% paraformaldehyde and then these tissues were sealed in paraffin and cut to 5 *μ*m thickness. The primary antibody, anti-insulin (purchased from Wuhan Boster Bio-engineering Limited Company [1:200]), was incubated with tissues and then probed with a secondary antibody. After rinsing, the Elivison two-step method was performed for immunohistochemical staining. An optical microscope was used to observe the slices (400×).

### 2.6. Measurement of Antioxidant Status

Glutathione peroxidase (GSH-PX), superoxide dismutase (SOD), and methane dicarboxylic aldehyde (MDA) in liver homogenates of mice were measured by using assay kits. The GSH-PX assay kit (A005), SOD assay kit (A001-1), and MDA assay kit (A003-1) were purchased from the Nanjing Jiancheng Bioengineering Institute, Nanjing, China. In accordance with the assay kit guide book, the absorbance of samples was determined at 412 nm for GSH-PX, 550 nm for SOD and 532 nm for MDA at the end of the reaction.

### 2.7. Quantitative Real Time-PCR Analysis

The extraction of pancreas and livers in total RNA, determination of RNA, and synthetization of the primer gene sequences of PI3K, PDK-1, AKT, FoxO1, and *β*-actin were carried out as reported previously [26]. Primer sequences are listed in Table 1, and they were synthesized by Sangon Biotech (Shanghai) Co., Ltd., China.

The reverse transcription reaction system was assembled on ice according to the kit. There are 250–300 ng mRNA for reverse transcription reactions. The reaction system was set to 37°C for 15 min. The reverse transcriptase was then inactivated by heating to 85°C for 5 sec, and the cDNA was obtained. There is 2 *μ*L cDNA for PCR reaction. PCR reaction conditions were as follows: 94°C for 4 min, 94°C for 20 sec, 60°C for 30 sec, and 72°C for 30 sec. Thirty-five cycles were conducted from the second stage. Using the different concentrations of cDNA as template, the standard curves of the experimental genes and the internal reference gene were generated. The data were calculated according to the following formula:* (T) = a × *lg*(copy) + b*; values of* a* vary among the different genes and PCR fragments.

### 2.8. Protein Expression by Western Blotting

Western blotting was done according to the methods described by Afrin* et al.* [28]. In the liver samples, the all-protein concentration was measured by the bicinchoninic acid method. To analyze the protein levels, protein extracts of the same quality were separated by using 7.5–15% SDS polyacrylamide gel electrophoresis. Then the protein was transferred from electrophoresis membranes to nitrocellulose membranes. Nitrocellulose membranes were blocked by 5% skimmed dry milk. Primary antibodies against PI3K (BM5187), AKT (BM4808), pAKT (BM4744), and GAPDH (BM1623) were purchased from Wuhan Boster Bio-Engineering Limited Company; primary antibody pFoxO1 (9461T), FoxO1 (2880T), and Lamin A (2032S) were from Cell Signaling Technology. Among primary antibodies, GAPDH was used at a dilution of 1:1300 and incubated overnight at 4°C. The following day, the membranes were washed with TBST three times each for 5 minutes. The secondary antibodies, which were bound to chemiluminescence developing agents, were used at a dilution of 1:5000 and incubated for 1.5 h. After that, the membranes were washed again with TBST four times each for 5 minutes. Films were scanned, and the results were analyzed with Quantity One.

### 2.9. Cell Fractionation

The cytoplasmic and nuclear protein was collected using nuclear and cytoplasmic protein extraction kit (Boster, AR0106). The liver was cut into small pieces and made to be tissue homogenate with ice-cold buffer A. Buffer B was added to the samples followed by 1 min centrifugation. The cytoplasmic supernatant was then collected. 100 mL ice-cold buffer C was added to centrifugal precipitate which containing 1 mL DTT and 5 mL 100 mM PMSF per mL Buffer C, 5 *μ*L protease inhibitor. Then the supernatant was centrifuged at 4°C for 16 000 g, 10 minutes, and transferred to ice-cold clean micro-centrifugal tube as soon as possible to obtain nucleoprotein. Protein levels in the cytoplasmic and nuclear extracts were detected following the western blot procedure.

### 2.10. Statistical Analysis

The experimental data were expressed as the mean ± SD and were analysed with Origin 8.5 software. The one-way ANOVA was used to evaluate the difference between multiple groups, with P < 0.05 considered as significant and P < 0.01 as very significant.

## 3. Results

### 3.1. Effect of MBBP on the Body Weight of Mice and the Coefficients of Organs to Body Weight

During the experiment, the weight of the normal mice increased steadily and the speed of weight gain slowed down a little within third weeks. The body weight of the all five groups increased in the same trend during the first two weeks. On the 14th day, the mice in the model group and the mulberry powder feeding group were injected with STZ. After that the weight of the mice in the model group has a sharp drop. The weight of mice in the 2.5% and 5% doses of mulberry powder feeding group also decreased, indicating that STZ could significantly affect the body weight, growth, and development of mice. After STZ injection, there was a significant difference in body weight between the three mulberry powder feeding groups. There was no significant difference between the 10% dose group and the normal group. As mice consume less MBBP, they lose weight faster. It indicated that the mulberry powder feeding could inhibit the weight loss of mice caused by STZ in a dose-dependent way.

**Figure 1:**
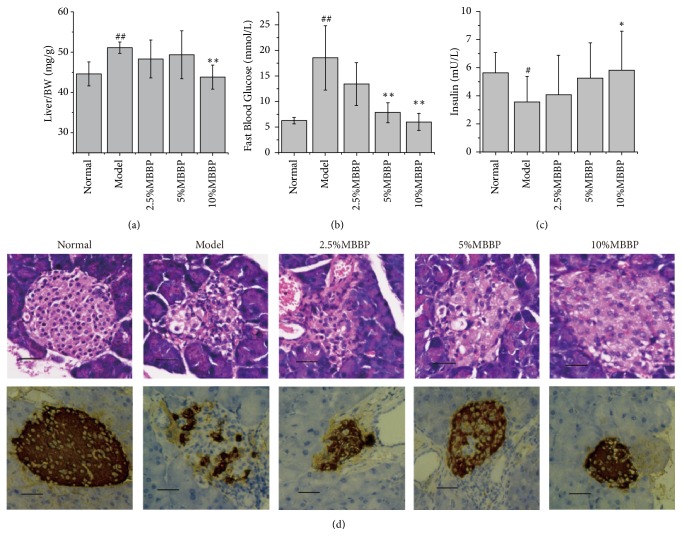
(a) The effect of MBBP on the coefficients of Liver to body weight; (b) The effect of MBBP on FBG after STZ injection; (c) the insulin levels of five groups (all data were presented as mean ± sd, the animal number, normal, n = 10; model, n = 7; each MBBP treat group, n = 8; # p <0.05, ## p <0.01 versus normal group; *∗* p<0.05, and *∗∗* p<0.01 versus model group); (d) the effect of mulberry branch bark powder on pathologic tissue in the pancreas and insulin immunohistochemistry of pancreas in mice. Scale bar: 50 *μ*m.

The coefficients of the liver to body weight are expressed as milligrams (wet weight of tissues)/grams (fasted body weight). As shown in Figure 1(a), the coefficients of liver in the model group were obviously higher than that of the normal group (*p < *0.01 or* p < *0.05); those of the model group reached 51.1 mg/g, indicating that liver appeared to have pathological changes. The reduction of these coefficients was observed in MBBP-treated groups compared with the model group; particularly the coefficient of the liver was the dose-effect manner in MBBP-treated groups. These data illustrated that oral administration of MBBP could reduce liver pathology changes induced STZ.

### 3.2. Effect of MBBP on the Fast Blood Glucose

After STZ injection, the FBG level was measured on 21th days after fasting 12 h. As shown in Figure 1(b), the mean FBG level of the model group continually rose and was obviously higher than that of the normal group (*p < *0.01 or* p < *0.01). The 2.5% MBBP-treated group was also higher than that of the normal group (*p < *0.05 or* p < *0.01). Compared with the model group, the FBG level in the 10% MBBP-treated group was lowest (*p < *0.01), followed by the 5% MBBP-treated group (*p < *0.05). On the 21th day, the dose-effect manner was more obvious to be observed in the MBBP-treated groups. These results showed that MBBP had an effect on controlling the exaltation of the FBG level induced by STZ.

Generally, diabetes was defined as FBG level ≥ 11.1 mmol/L. According to this standard, the incidence of diabetes was also counted. There were no diabetic mice in the normal group, the incidence of diabetes reached 75% in the model group, and there was a downward trend accompanied by the dose-effect manner in MBBP-treated groups, 66.7% for 2.5% MBBP group, 12.5% for 5% MBBP group, and 0% for 10% MBBP group. The incidence in the 10% MBBP group was lowest in the MBBP-treated groups. Consequently, the results showed that MBBP could efficiently decrease the incidence of type 1 diabetes induced by STZ.

### 3.3. Effect of MBBP on Serum Insulin and Insulin Secretion

The mean value calculated at the end of the experiment is shown in Figure 1(c). The mean value of insulin level was obviously lower in the model group than that of the normal group (*p < *0.05). The insulin level is 1.69 and 2.26 mU/L higher in the 5% and 10% MBBP-treated groups compared with the model group, respectively (*p < *0.05). The results represent the decreased secretion of insulin due to the damage of pancreatic *β*-cells induced by STZ. Due to the intake of MBBP, the occurrence of this process was mitigated.

The pancreas is an important organ that regulates blood glucose and insulin secretion. This experiment used H&E staining to evaluate the protective effect of MBBP on the pancreas, as shown in Figure 1(d). The pancreas of the normal group showed normal structure. The pancreas of the model group showed minute inflammatory cells infiltration, slight interstitial proliferation, angionecrosis, and tiny congestion of vascellsum and prominent vasodilation. The extent of pancreas injury in MBBP-treated groups was better than that of the model group and was in a dose-effect manner. These pictures indicated that MBBP could effectively prevent pancreas injury induced by STZ to a certain degree.

To study insulin secretion in pancreas tissue, the immunohistological method was used to observe the state of insulin secretion (Figure 1(d)). The insulin was dyed dark brown, and a large dark brown area was observed in the normal group. Insulin secretion was barely visible in the model group. Insulin secretions in MBBP-treated groups were dramatically better than that of the model group, particularly notable in the dark brown area of the 10% MBBP-treated group. These pictures show that MBBP had an outstanding effect on preventing the decline of insulin secretion.

### 3.4. Measurement of Antioxidant Capacity

To evaluate the influence of MBBP on antioxidant capacity, the levels of T-SOD, GSH-PX, and MDA were investigated. The results in Table 2 show that the pancreas injury was correlated with the depletion of antioxidant status (SOD and GSH-PX) and excessive accumulation of one final oxidation product (MDA). Compared with the normal group, the MDA level in the liver was significantly increased (*p < *0.01), and the activities of antioxidant enzymes (T-SOD, GSH-PX) were significantly decreased (p < 0.01) in the model group. However, the situation changed with MBBP treatment before STZ injection. MDA level was significantly decreased (*p < *0.05 or* p < *0.01) and the activities of antioxidant enzyme (T-SOD, GSH-PX) were significantly increased (*p < *0.01) in MBBP-treated groups compared with the model group.

### 3.5. Effect of Expression of MBBP on PI3K/ATK/FoxO1 Signal Pathway Key Protein

**Figure 2:**
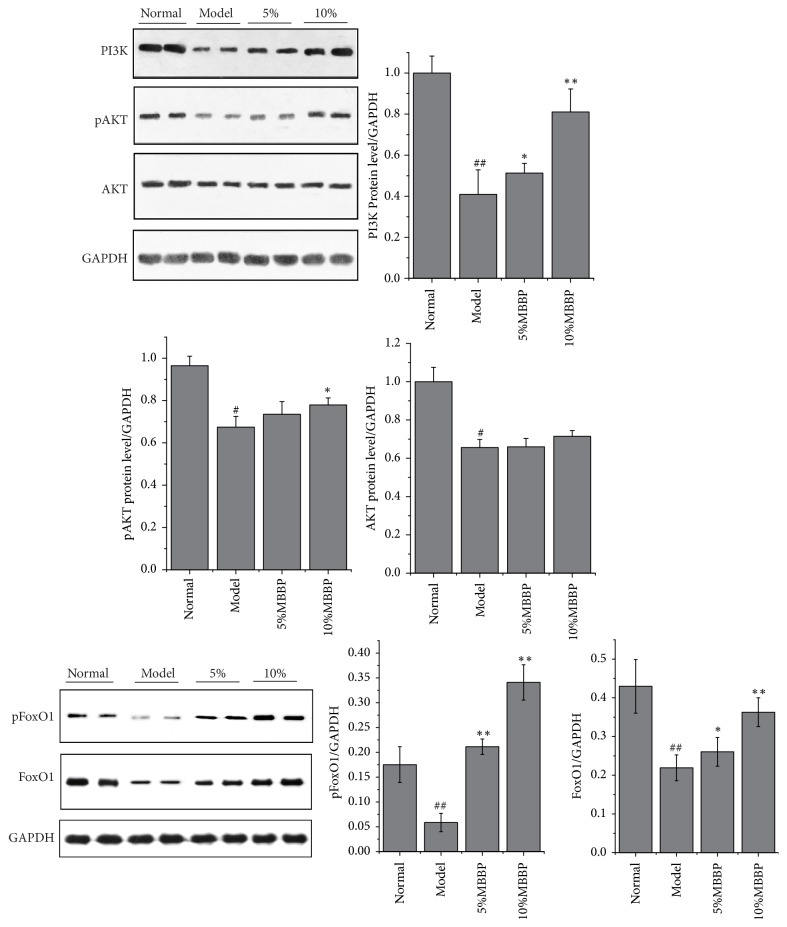
Protein levels of PI3K, p-AKT, AKT, pFoxO1, and FoxO1, in the liver assayed by Western blot. Density values were normalized to GAPDH levels (ALL data were presented as mean ± sd, n = 4, # p < 0.05 and ## p < 0.01 versus normal group; *∗* p < 0.05 and *∗∗* p < 0.01 versus model group).

**Figure 3:**
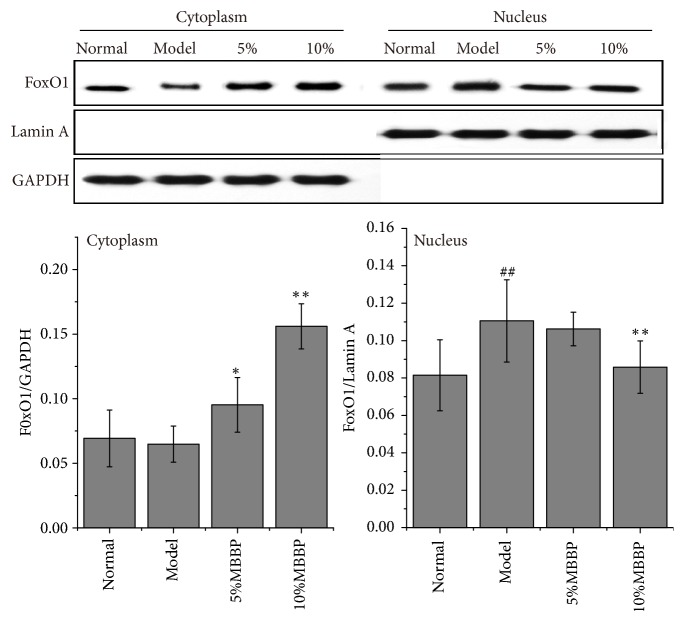
Protein levels of FoxO1 in the cytoplasm and nucleus, assayed by Western blot. Density values were normalized to GAPDH and Lamin A levels (all data were presented as mean ± sd, n = 3, # p < 0.05 and ## p < 0.01 versus normal group; *∗* p < 0.05 and *∗∗* p < 0.01 versus model group).

The present experiment further investigated PI3K/AKT/FoxO1 signal pathway. The expression levels of PI3K, AKT, phosphorylated AKT, FoxO1, and phosphorylated FoxO1 protein in liver tissue were detected by Western blotting (Figure 2) in all mice. The data in Figure 2 showed that the expression of those proteins had an upward trend in MBBP-treated groups compared with the model group; the expression levels of PI3K, pAKT, and pFoxO1 in 10% MBBP-treated group showed a significant rise. Although the level of FoxO1 increased, the ratio of pFoxO1 to FoxO1 was also high in MBBP treatment group when compared with that in model group. The FoxO1 in cytoplasm and nucleus also have been tested and presented in Figure 3. The expression levels of FoxO1 in cytoplasm increased significantly in 10% MBBP treated group comparing with that in model group. Correspondingly, the FoxO1 level in nucleus was observed decreased with MBBP treatment in dose dependent manner.

### 3.6. Effect of MBBP on the Gene Expression of Signal Pathway PI3K/AKT/FoxO1

**Figure 4:**
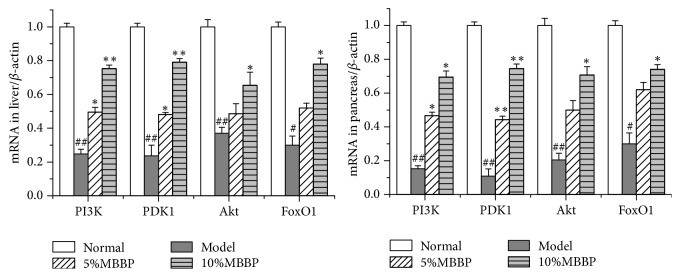
Preventive effect of MBBP to PI3K, PDK-1, AKT, and FoxO1 mRNA expression in the liver and pancreas (all data were presented as mean ± sd, n = 3, # p < 0.05 and ## p < 0.01 versus normal group; *∗* p < 0.05 and *∗∗* p < 0.01 versus model group).

Gene expression of PI3K, PDK1, AKT, and FoxO1 in the pancreas and liver was analysed in the study. As shown in Figure 4, the expression of each gene in the normal group was obviously higher than that of the model group (*p < *0.05 or* p < *0.01). Gene expression in MBBP-treated groups obviously increased (*p < *0.05 or* p < *0.01) compared to the model group, and the phenomenon presented a dose-effect manner; the expression in the 10% MBBP-treated group was obviously higher than that of the model group (*p < *0.05 or* p < *0.01). These data indicate that MBBP could increase the expression of PI3K, PDK1, AKT, and FoxO1 which were lower in pancreas and liver of mice of model group.

## 4. Discussion

In this study, the pancreas injury of mice was induced by STZ, and the dysfunction of insulin secretion and hyperglycaemia were observed due to damaged *β* cells of pancreas. With preventive administration of branch bark powder in mice, the extent of pancreatic injury and hyperglycaemia were observed relieved.

Under normal circumstances, the pancreas releases insulin into the bloodstream when the level of blood glucose is high. With damaged *β* cells of pancreas due to STZ injection in mice, hyperglycaemia and low insulin level, namely, insulin resistance, were observed in non-MBBP feed group (model group). Several markers of lipid metabolism were also observed to be abnormal during the process. The total triglycerides (TG) level was significantly increased and the high-density lipoprotein cholesterol (HDL-C) level was significantly decreased in model group. On the other hand, hyperglycaemia can lead to glycosylation of antioxidant enzymes [29, 30], and the decreased activity of antioxidant enzymes such as Superoxide Dismutase (SOD) and Glutathione peroxidase (GSH-Px) were also observed. Malondialdehyde (MDA) is one of the final products of polyunsaturated fatty acids peroxidation in the cells. An increase in free radicals causes overproduction of MDA [31]. Thus the increased MDA level of the mice in model group also indicates the imbalance of antioxidant capacity. Compared with these results of model group, the MBBP feed groups showed opposite tendency in a dose-effect manner. The levels of blood glucose, insulin, TG, HDL-C, MDA, SOD, GSH-Px, and body weight were all observed to significantly change in the opposite direction compared with the model group. After only two weeks of MBBP feeding, the phenotypic detection results of these mice in MBBP feed groups presented improved ability to resist STZ damage.

In vivo, insulin resistance in the liver appears to result from the failure of the insulin to inhibit hepatic glucose production. The PI3K/AKT signalling pathway plays a role in glucose storage and uptake in the liver [32]. FoxO1, also known as FKHR, is a transcription factor in the downstream of this insulin signalling pathway [33, 34]. Insulin causes the activation of PI3K, which subsequently phosphorylates AKT. The p-AKT then phosphorylates FoxO1, causing nuclear exclusion. The translocation of FoxO1 from the nucleus to the cytoplasm suppresses the expression of FoxO1 gluconeogenic targets, phosphoenolpyruvate carboxylase (PEPCK), and glucose-6-phosphatase (G6Pase) in nuclear [35–37]. Proteins of PEPCK and G6Pase are rate-limiting enzymes for gluconeogenesis. In light of these reports, our studies show that MBBP feed resulted in enhancement of the PI3K, phosphorylation of both AKT and FoxO1. This will prolong insulin's inhibitory effect on hepatic glucose production accompanied by suppression of the expression of PEPCK and G6P in the liver. These results are in accord with the reduced blood sugar in MBBP treat groups.

On the other hand, the activation of the PI3K/AKT/FoxO1 pathway was also found in pancreas of MBBP group mice. According to the existing paper, FoxO1 also play an important role in apoptosis and cells cycle regulation. Increasingly evidence showed that disruption of AKT/FoxO1 signalling might cause the apoptosis of *β* cells. Inhibition of FoxO1 (phosphorylates FoxO1 and translocate to cytoplasm) could promote *β* cells survival [38, 39]. This is in accord with our results of histopathological observation. But the exact molecular mechanism of apoptosis regulation has not been clarified. Therefore, it would be interesting to conduct further studies to detect downstream gene target of FoxO1.

In conclusion, our results indicate that MBBP feed attenuates STZ induced dysfunction and injury in pancreatic *β* cells for its beneficial metabolic effects. The PI3K/AKT/FoxO1 signalling is involved in the protective effect of MBBP by activation of AKT, inhibition, of FoxO1. Thus, MBBP improve the tolerance of pancreas to the injury of STZ and may be promising for the prevention of pancreas injury.

## Figures and Tables

**Table 1:** Primers of reverse transcription-PCR analysis for genes.

Gene	Primer sequences (5′ to 3′)	Product length (bp)
AKT-F	TGTCTGCCCTGGACTACTTGC	166
AKT-R	GGCGTTCCGCAGAATGTC	
PI3K-F	AAGCCATTGAGAAGAAAGGACTG	176
PI3K-R	ATTTGGTAAGTCGGCGAGATAG	
PDK-1-F	CTGGGCGAGGAGGATCTG	132
PDK-1-R	CACAGCACGGGACGTTTC	
Foxo1-F	AGTGGATGGTGAAGAGCGTG	137
Foxo1-R	CTTTCCAGTTCCTTCATTCTGC	
*β*-Actin-F	GAGACCTTCAACACCCCAGC	263
*β*-Actin-R	ATGTCACGCACGATTTCCC	

**Table 2:** The effect of MBBP on antioxidant capacity.

Groups	T-SOD (mmol/L)	GSH-PX (mmol/L)	MDA (mmol/L)
normal group	141.35±8.08	1006.44±145.91	0.859±0.028
Model group	129.30±3.15##	900.53±76.79##	1.459±0.224##
2.5% MBBP	136.21±9.33	1092.03±85.38	1.097±0.202
5% MBBP	144.49±14.54*∗*	1097.48±120.36*∗*	0.987±0.027*∗*
10% MBBP	161.36±18.28*∗∗*	1114.12±85.16*∗∗*	0.963±0.121*∗∗*

#*p*<0.05 and ##*p*<0.01 versus normal group; *∗p*<0.05 and *∗∗p*<0.01 versus model group.

## Data Availability

All data used to support the findings of this study are included within the article. All data created during this research are available from the corresponding author upon request.
